# Profiling the Occupational Injuries Sustained by Custody Officers: A Systematic Review

**DOI:** 10.3390/healthcare12232334

**Published:** 2024-11-22

**Authors:** Louis Reilly, Jessica Chan, Thevanthi Thevanesan, Robin Orr, Jay Dawes, Robert Lockie, Elisa Canetti, Ben Schram

**Affiliations:** 1 Faculty of Health Sciences and Medicine, Bond University, Robina, QLD 4226, Australia; louis.reilly@student.bond.edu.au (L.R.); jessica.chan@student.bond.edu.au (J.C.); rorr@bond.edu.au (R.O.); ecanetti@bond.edu.au (E.C.); 2 Tactical Research Unit, Bond University, Robina, QLD 4226, Australia; 3 Department of Health and Human Performance, Oklahoma State University, Stillwater, OK 74074, USA; jay.dawes@okstate.edu; 4 Tactical Fitness and Nutrition Lab, Oklahoma State University, Stillwater, OK 74078, USA; 5 Department of Kinesiology, California State University, Fullerton, Fullerton, CA 92831, USA; rlockie@fullerton.edu

**Keywords:** law enforcement, workplace musculoskeletal disorder, occupational health, prison officer

## Abstract

Background/Objectives: Custody officers (CO) are often exposed to workplace hazards when monitoring prisoners, managing prisoners’ recreational time, or searching for contraband, yet research into their injuries is limited. This review aimed to identify, appraise, and synthesise research investigating injuries in CO. Methods: Following the Preferred Reporting Items for Systematic Reviews and Meta-Analyses protocol and registration with the Open Science Framework, a systematic search of five databases (PubMed, ProQuest, Embase, CINAHL and SportDiscus) using key search terms was conducted. The identified studies were considered against eligibility criteria, with the remaining studies critically appraised using the appropriate Joanna Briggs Institute checklist. Results: From the 975 identified studies, eight studies (mean critical appraisal score = 69 ± 25%) remained to inform the review. The incidence of fatal injuries ranged from 0.027 to 0.03 per 1000 full-time employees (FTE), whereas that of non-fatal injuries ranged from 15.9 to 44.0 per 1000 FTE. CO aged 31+ years were the most likely to experience injuries (22–44%). Male CO were more commonly injured than female CO in both fatal injuries (male = 89%, female = 11%) and non-fatal injuries (male = 73–74%, female = 26–27%). Assaults (11.5–38%) and slips/trips/falls (23.2–25%) were found to be the most common causes of injuries. The upper extremity was the most commonly injured body part (26–30%), with musculoskeletal sprains and strains (30–60.2%) the most common types of injury. Conclusions: CO injury profiles are similar to those reported in general-duty police officers. As such, musculoskeletal conditioning, reconditioning, and fall prevention practices employed in law enforcement may serve as an initial approach to risk mitigation in this population.

## 1. Introduction

Custody officers (CO), also known as correctional officers or prison officers, are trained professionals who play a crucial role in ensuring the safety, security, and efficiency of correctional centres and facilities [1]. Their responsibilities include monitoring prisoner conduct and behaviour; preventing prisoner disturbances or escapes; overseeing prisoners during work assignments; managing prisoners’ recreational time, sport activities, and meals; conducting searches of prisoners and cells for weapons or contraband; patrolling assigned areas; reporting rule breaches by prisoners; and coordinating the transport of prisoners between courts and prisons [2]. According to the most recent report generated by the Australian Institute of Health and Welfare, there are currently 116 prisons in Australia [3], with 24,900 CO employed, the majority of which are male (73%) with an average age of 46 years [4]. In the United States (U.S.), there are a total of 1566 state prisons [5], employing 363,250 CO, with slightly lower male representation (68%) when compared to Australia and a younger average age of 40 years [6,7].

Generally, for most countries, prisons fall under four common types of security levels: minimum-, low-, medium-, and high-/maximum-security institutions [8,9]. Minimum-security institutions usually have limited or no perimeter fencing, relatively low staff to inmate ratios, and dormitory housing. In contrast, high-security prisons have highly secured perimeters, multiple- and single-occupant housing, and the highest staff to inmate ratios [9]. These institutions vary depending on the seriousness of prisoners’ offences and the institutions’ security features, such as the presence of external patrols, towers present, security barriers (walls and fencing), detection devices, types of housing, and staff to inmate ratios [9]. Minimal published evidence exists discussing the differences between the level of violence and the level of institution security in relation to injuries. However, Lincoln et al. [10] found that medium- and maximum-security institutions in the U.S. exhibit comparable rates of staff injury, but mixed-security (a combination of both medium and maximum) prisons exhibited significantly higher numbers of attacks than the two security levels individually [10]. 

The nature of the CO occupation entails significant physical demands and exposure to various workplace hazards, such as violent attacks and physical injuries due to overexertion, presenting risks often higher than typical working environments [11]. Due to these risks, CO, regardless of the institution’s security level, are highly susceptible to workplace injuries and health issues [12,13]. For example, in the U.S., the CO occupation is considered to have one of the highest rates of days away from work due to non-fatal injuries, with almost 446 injuries per 10,000 full-time workers [12,14], stemming primarily from assaults and violent acts, slips/trips/falls, and contact with objects [12,15,16]. These workplace injuries not only have a profound effect on the individual, but also affect their families, their colleagues, the institutions that they work for, and society at large. 

Due to the pressing challenges faced in this occupation, reports indicate high staff turnover rates and difficulties in attracting new recruits, with 75% of employers reporting a shortage of skills among the occupation [17]. Moreover, prison populations are on the rise in Australia, increasing by approximately 38% over the decade of 2012 to 2022 [3]. This is comparable to the populations of China’s prisons, which have the highest number of incarcerated individuals worldwide, totalling approximately 1.69 million people [18]. China showed an increase of approximately 20% in prisoners over a span of 17 years (from 2000 to 2017) [19]. This population trend adds additional stress and challenges to the profession as the ratio of prisoners to CO continues to increase at a disproportionate rate. Prison population increases, together with employer dissatisfaction and workplace dangers, highlight workplace challenges within this occupation. Noting these challenges, research on the well-being and safety of CO has been limited, making evidence-based approaches to improve safety precautions, policy changes, and interventions difficult. 

While a systematic review has profiled the injuries sustained by law enforcement officers (LEO) [20], no such review has been conducted in a CO population. This systematic review seeks to improve the understanding of the injury profiles of CO to bridge the gap in knowledge. Through a detailed profile of workplace injuries, evidence-based and targeted safety measures, training, or policy changes to enhance safety in corrections facilities can be established. As such, the aim of this review was to identify, critically appraise, and synthesise key findings from the recent literature investigating musculoskeletal injuries sustained by CO, in order to develop a profile of the injuries experienced by this unique population. 

## 2. Materials and Methods

### 2.1. Protocol and Registration

This systematic review followed the Preferred Reporting Items for Systematic Reviews and Meta-Analyses (PRISMA) guidelines [21]. The systematic review protocol was registered with the Open Science Framework [22] prior to the commencement of this research. 

### 2.2. Information Sources and Search Strategy

This review sourced literature through a search of five academic databases, PubMed, ProQuest, Embase, the Cumulative Index to Nursing and Allied Health Literature (CINAHL), and SportDiscus. A systematic search strategy using carefully selected key words, search terms, and Medical Subject Headings (MeSH) terms, where appropriate, was employed. These key words and terms were identified through an initial search on PubMed and Google Scholar to identify studies already known to the authors. Working with the university librarian, the key search terms were enhanced and the search strategy validated. Once these terms and the search strategy were finalised for the PubMed database (Table 1), the strategy string was translated for use in the other databases (Embase, CINAHL, ProQuest, and Sport Discus) using the Polyglot search translator [23] (Supplementary File S1). Other search strategies include the citation searching of included articles, as well as the identification of research and grey literature on websites through employing terms in web browser search engines. All identified results were exported to EndNote [24] and underwent eligibility screening against a priori established eligibility criteria. 

### 2.3. Eligibility Criteria

The eligibility criteria were shaped using the Joanna Briggs Institute (JBI) Population (or participants)/Concept/Context (PCC) model framework [25], where the population of interest was CO and other nomenclature associated with this occupation (e.g., prison officers, correctional institution officers, custody officers, etc.). The concept of interest was injury data that pertained to fatal or non-fatal occupation-related injuries. The context included any geographical location, with reports in English (or accurately translatable to English) and considering CO of any sex, race, or age. Based on this framework, the inclusion and exclusion criteria (see Table 2) were established, with each identified article subjected to these criteria.

### 2.4. Selection Process/Screening

The screening of the articles was performed by two independent reviewers using a staged process after the search results were imported into EndNote. The first process involved the removal of duplicates, which was performed using the EndNote duplicate removal function. The remaining literature were screened by title and abstract, and those articles clearly not related to the topic were removed. The remaining articles were then screened against the inclusion and exclusion criteria by two authors (L.R. and J.C.) independently. A third author (T.T.) was used for consensus regarding any discrepancies/differences. 

### 2.5. Quality Assessment 

Following the completion of the literature screening, all remaining studies that met the inclusion criteria but failed to meet the exclusion criteria underwent a critical appraisal assessment. This assessment was performed with the appropriate JBI tool for risk of bias assessment (Checklist for Analytical Cross-Sectional Studies) [26]. This checklist consists of eight questions, with no modifications made to the checklist for this review. Critical appraisal was performed independently by two authors (L.R. and J.C.). Each article received a total score out of eight, with the final score converted to a percentage score for grading. A score of “1” was given for a “yes” answer and a score of “0” for an “unclear” or “no” answer. Grading categories, based on previous research in a law enforcement population [20], were used to classify the studies as poor (<45.4%), moderate (45.5–61%), or good (>61%) in relation to their risk of bias assessment. Once all studies were scored, Cohen’s Kappa was used to determine the level of agreement between the reviewers with scoring for the levels of agreement based on the works of Viera and Garrett [27]. Following the scoring and the determination of the levels of agreement, the reviewers met to discuss their scoring and came to an agreement on the final scores, with any discrepancies resolved by a third reviewer (T.T.).

### 2.6. Data Extraction and Management

The extraction of data involved two authors (L.R. and T.T.). The table for data extraction was piloted by both authors independently using one paper. Following this process and table heading consensus, key variables and the main findings of interest were identified and extracted into a table. The items extracted from the literature included the study details (author and year), study demographics (age groups of data, officer sex, types of injury examined, setting, and types of officers involved), and any data relating to injuries (injury prevalence, causes of injury, body parts injured, and nature of injury). This information was extracted and tabulated. 

### 2.7. Data Analysis and Synthesis

For this review, and due to the known lack of current literature on the topic, a broad approach was used to profile CO injuries. However, due to variations in the research methodologies and data-reporting approaches within the included articles, a meta-analysis could not be performed. Instead, as per previous research approaches [20,28], the data were synthesised and analysed for any information regarding injury rates, the body parts injured, the types of injury, and the causes of injury. Injury incidence rates were calculated and transformed in the number of injuries per 1000 full-time employees (FTE) for both non-fatal and fatal injuries. Naturally emerging themes were used to inform the results and discussion.

## 3. Results

**Figure 1:**
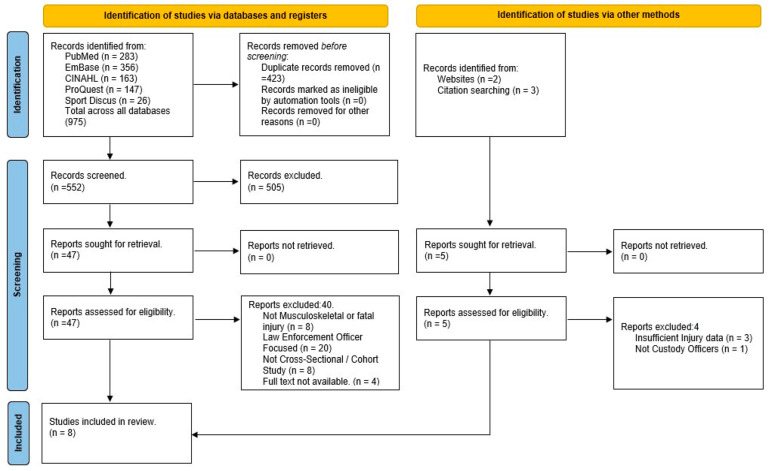
PRISMA flow diagram detailing the article identification, screening, and selection process [21].

The results of the identification and screening process were represented in a PRISMA flow diagram (Figure 1). The initial search identified 975 records, along with five documents that were identified via other methods and did not appear among the search strategy (two via websites and three via citation searching). The removal of 423 duplicate records left 552 records to be reviewed by title and abstract to identify records clearly not relevant to the study (e.g., dealing with individuals who have mental illnesses: the Crisis Intervention Team (CIT) in law enforcement [29]). The resulting 52 records were reviewed in detail and considered against the inclusion and exclusion criteria. Following this process, eight studies were left to inform the review, with the remaining studies and their reasons for exclusion presented in Figure 1.

### 3.1. Critical Appraisal

The final scores and ratings of quality for the critical appraisal of each of the included studies using the JBI Checklist for Analytical Cross-Sectional Studies are shown in Table 3, with the final raw scores shown in Supplementary File S2. The Cohen’s Kappa analysis indicated that there was “moderate agreement” between the reviewers (k = 0.424). Regarding the methodological quality scores of the included studies, five studies were graded as “good” [12,15,16,30,31], one study was graded as “moderate” [32], and two studies were graded as “poor” [10,33]. The mean score (±standard deviation) among the included studies was 69 (±25)%. Item 6 of the JBI tool, which refers to strategies dealing with confounding factors, was the most common item receiving a low score, with seven of the eight studies scoring a “no” for this question. This may indicate cofounding bias within the literature, where the effect of exposure may be mixed with extraneous risk factors.

### 3.2. Incidence and Prevalence of Injury

The incidence of injury was reported by four studies [10,12,15,30] and the prevalence by one study [31]. Of these studies, two reported fatal injury rates [12,30], two reported non-fatal injury rates [12,15], one reported an incidence rate of injury due to weapons [10], and one reported the prevalence of chronic pain [31]. The reported incidence of fatal injuries ranged from 0.027 [12] to 0.03 fatal injuries per 1000 FTE [30]. For non-fatal injuries, the incidence rates were between 15.9 and 44 injuries per 1000 FTE [12,15]. The incidence of injuries due to weapon attacks was 1.0 (0.66–1.28, 95% CI) per 1000 FTE [10]. Chronic pain was reported at a prevalence of 45.4% among CO [31]. 

### 3.3. Demographics of Injured Persons

Of the included articles, five were located in the United States [10,12,15,30,32], two were located in Australia [16,33], and one was located in Canada [31]. Three articles reported trends among the age groups of injured CO [12,15,32]. Konda et al. [12] reported that the eldest categorised age group (45 years and older) was the most likely to receive a fatal and non-fatal injury (44% and 22%, respectively), while the results of Holloway-Beth et al. [15] suggest that the largest proportion of non-fatal injuries was for those aged 31–40 (36%) years. When considering the mean age of those experiencing fatal attacks by knife, the reported mean age was 40.5 years, with 33.3% of the fatal knife attacks occurring among those aged between 45 and 49 years, being the most common [32]. In terms of the facility security level, the research by Lincoln et al. [10] found that maximum- and medium-security facilities exhibited similar rates of injury by weapon, at rates of 0.6 and 0.5 per 1000 FTE, respectively. Meanwhile, mixed-security prisons exhibited a significantly higher level of injury by weapon, at 2.0 per 1000 FTE [10]. 

Two studies compared the proportions of injuries between male and female CO, with both studies reporting similar findings [12,15]. For non-fatal injuries, 27% and 26% of the injuries occurred to female CO and 73% and 74% to male CO [12,15]. For fatal injuries, 11% of the fatalities occurred to female CO and 89% to male CO [12]. 

### 3.4. Causes of Injury

Among the eight articles, five [12,15,16,30,33] examined the different causes of injury. For fatal injuries, two studies reported that 40% [12,30] of the fatalities were caused by transportation-related incidents, while homicides were reported at 25% [12] and 29% [30] as the second-highest causes. Causes of non-fatal injury were reported by three studies [12,15,16]. Commonly mentioned causes included assaults and violent attacks, slips/trips/falls, and contact with objects or equipment. The assault and violent attack values ranged from 11.5% [16] to 38% [12], slips/trips/falls ranged from 23.2% [16] to 25% [15], and contact with objects or equipment ranged from 18% [12] to 21.8% [16]. Finally, Larney and Dolan [33] specifically measured the incidence of needlestick injuries, finding an injury rate of 10%. This figure differed from that of Ngwenya [16], who reported a much lower rate of 2.7%. 

### 3.5. Nature of Injuries and Body Parts Injured

Among the eight articles, the nature of the injuries was described in two studies [12,16], with both reporting that sprains and strains were the most common types of injury sustained, accounting for 30% [12] and 60.2% [16] of the injuries. Four out of the eight articles described the body parts injured [12,15,31,32]. The most commonly injured were reported as the upper extremities, ranging from 26% [15] to 30% [12] of all reported injuries. Chenpanas et al. [32] identified areas of fatal knife assaults, with the neck, chest, and shoulder sites being commonly targeted and also deemed the most vulnerable body parts. Carleton et al. [31] reported that the lower back was the primary body site associated with chronic pain (26.1%). 

## 4. Discussion

The aim of this systematic review was to identify, critically appraise, and synthesise key findings from the recent literature investigating musculoskeletal injuries sustained by CO. The main findings of this review were compiled into five naturally emerging themes for ease of synthesis: (1) injury incidence; (2) demographics of injury, (3) common causes of injury; (4) commonly injured body parts; and (5) nature of injury. In this review, the injury incidence rates ranged from 15.9 to 44.0 per 1000 FTE for non-fatal injuries and from 0.0027 to 0.003 per 1000 FTE for fatal injuries. Older male employees were found to be the most commonly injured. The most common causes of injury were assaults and slips/trips/falls, whereas, for fatalities, the cases were commonly homicides and transportation-related. The most commonly injured body sites were the upper extremities, and the most common types of injuries were sprains/strains. 

Regarding the incidence of injuries, it was found that non-fatal injuries ranged from 15.9 to 44.0 per 1000 FTE. These prevalence rates fall within those of the general population regarding work-related incidents in both Australia (35 injuries per 1000 FTE [34]) and the U.S. (27 per 1000 FTE [35]). However, these rates are notably lower than those reported in other tactical populations. A systematic review of firefighters suggests an injury incidence rate of 177 firefighters per 1000 FTE [28]. In law enforcement, these figures are notably higher, ranging from 240 to 2500 officer injuries per 1000 FTE [20]. While the methods of reporting injuries may influence the findings [36], the potential second-order consequences of injuries to CO, as opposed to the general population, warrant consideration. In CO, the leading cause of injury is an assault by an offender who is imprisoned; as such, if an officer is injured during an altercation with an inmate, other potential officers, or potentially community members (if occurring during a transport), could be placed at risk.

Older males suffered the majority of both the fatal and non-fatal injuries in CO. These findings are not unexpected given the that the CO occupation is generally composed of male officers in this age demographic [4,6,7]. Considering this, research investigating age as a risk factor for injuries like sprains and strains is conflicting and can be dependent on the body site injured [37,38]. For example, a lower age has been found to be a risk factor for ankle sprain injuries [37], while a higher age has been found to be a risk factor for knee ligament injuries [38]. A final consideration may be in the attitudes of inmates who could be more likely to attack an older CO. This supposition warrants some support given that assaults are the leading causes of injury in this population. 

Among the included studies, the most common causes of injury presented a similar pattern, with assaults, slips/trips/falls, and contact with an object as the more commonly described leading causes of non-fatal injuries. Assaults, as a cause of injury, ranged between 11.5% and 38%. These findings are lower than, or similar to, those for LEO, which ranged between 21.3% [39] and 61.67% [20] of the recorded injuries classified as assaults from a non-compliant offender. A potential reason for these higher rates in law enforcement could be the nature of their duties, where officers have been described as “street-level bureaucrats”, handling violent situations, negative attitudes, and threats from citizens [40]. Conversely, slips/trips/falls were more frequently reported in the studies informing this review (23.2% to 25%) than those found in a law enforcement population (16%), although these results were similar to those reported in military (21.4% [41]) and firefighter (18–21.3% [28]) populations. With all of these tactical populations required to wear and carry loads consisting of essential equipment, it is of note that load carriage as a specific activity has been identified as a leading cause of injury in military personnel and could account for up to 30% of non-fatal occupational slip, trip, and fall musculoskeletal injuries [42]. Further, tactical populations, in general, tend to have higher levels of overweight and obesity than the general population. Ngwenya [16] reported that 74% of CO had a BMI that was classified as overweight (32.9%) or obese (41.1%), while Morse et al. [43] suggested higher rates of 83% (40.7% overweight and 43.3% obese). Law enforcement has reportedly similar rates [44], with similar BMIs [45], as do firefighters [46]. This is a concern given research showing that obese individuals have a higher probability of falls and ambulatory stumbling than normal-weight individuals [47,48,49].

Both studies that mentioned fatal causes of injuries followed a very similar pattern, where transportation-related events were the most common cause, reported at 40%, followed by homicides at 25–29% and suicides at 15% [12,30]. Research has investigated and compared the fatality causes in CO to other categories of LEO. It was found that CO had the lowest rates in terms of homicides but the highest in terms of suicides when compared to sheriffs, bailiffs, police detectives, guards, and police [30]. This trend was highlighted by the New Jersey Police Task Force, who found that CO had more than double the rate of suicide than police officers (34.8% for CO compared to 15.1% for police officers) [50]. The higher rates of suicide among CO may be explained by the high levels of work-related stress and psychological problems (such as anxiety, burnout, and frustration) in this occupation, which has also been noted as being relatively high in several studies [43,51,52]. This is a cause for concern and should be an avenue for future analyses in the CO population.

Regarding the body parts injured, upper-limb injuries were listed as the most common (26% to 30%). Of note, these findings are similar to those reported in LEO (32.95% to 42.42%), where the upper limb is considered the leading bodily site of injury. Assaults and altercations with non-compliant offenders, a leading mechanism of injury in both of these populations, present a potential reason for this bodily site being the most commonly injured. This supposition is supported by the lower limbs being reported as the leading sites of injury in firefighters [53] and military populations [54], which do not list assault as a mechanism of injury. 

In this review, strains and sprains were the most common types of injuries, ranging from 30% to 60.2%. These findings are similar to those of other tactical populations, with sprains and strains typically reported as the most common type of injury in law enforcement (42.3% to 94.6% [20]), fire and rescue (16% to 74%), the military (9.3% to 44% [55,56]), and paramedics (38–76% [57,58]). As such, the results of this review suggest that, like other tactical occupations, musculoskeletal injuries are most commonly sprains and strains. As such, means of mitigating or reducing the severity of, or optimising the rehabilitation for, these types of injuries gathered from other tactical organisations (e.g., the military) may be of benefit. One example is the early reporting and early treatment of these injuries [59].

While this systematic review presents an understanding of the injury profile for CO, it should be noted that there are several limitations that require consideration. Firstly, the amount of research available on the topic is very limited. This presents difficulty in being able to gain a comprehensive understanding of the topic. Additionally, this review may present some level of language bias [60]. Only literature that was published in English, or translatable to English, was included in the review, and only English databases were used. There is potential that literature in other languages could further add to the findings of this review. The limitations of the included studies include a high level of study heterogeneity, cofounding factor bias, and the fact that the included studies varied in both the methodologies and the characteristics that they measured and reported, including variations in the injury incidence, prevalence, or per unit exposure (/1000 FTE), which prohibited a meta-analysis. These limitations are typical of reviews investigating injuries in tactical populations [20,28]. The injury capture methods that were used included surveys, database analyses, and workers’ compensation analysis, which may affect the results due to the levels of reporting [36]. Future research should investigate how other demographic factors, such as the training level or years of service, may influence the injury rates within this population. Furthermore, while this review excluded mental health occupational injuries, these disorders threaten the well-being of correctional staff [61] and warrant further investigation. 

## 5. Conclusions

This review represents an initial step in recognising the attributes associated with work-related injuries and fatalities experienced by CO. The fatal injury incidence was found to be 0.027 to 0.03 per 1000 FTE, while that of non-fatal injuries was 15.9–44 per 1000 FTE. The data also showed that CO who were older males were most commonly injured, with assaults and slips/trips/falls being the most common causes of injury. The upper extremity was the body part found to be the most commonly injured, and sprains/strains were reported to be the most common types of injury. These findings bear similarities to those found in law enforcement populations, and, as such, the evidence-based injury mitigation strategies employed by law enforcement agencies may provide an initial step in minimising CO injuries. Future work on CO should focus on standardising the methods of gathering data and defining variables, such as standardising the injury data capture methods and definitions. 

## Figures and Tables

**Table 1:** Details of the search terms entered into PubMed.

Database	Date Searched	Search Terms
PubMed	7 September 2023	(“Correctional Facilities Personnel”[Mesh] OR “correctional officer *”[Title/Abstract] OR “Custody Officer *”[Title/Abstract] OR “Prison Officer *”[Title/Abstract] OR “correctional staff”[Title/Abstract] OR “Law Enforcement Officer *”[Title/Abstract] OR “Correctional Worker *”[Title/Abstract]) AND (“Wounds and Injuries”[Mesh] OR Injury[Title/Abstract] OR Injured[Title/Abstract] OR injuries[Title/Abstract] OR Trauma[Title/Abstract] OR Wound[Title/Abstract] OR fatal[Title/Abstract] OR events[Title/Abstract] OR nonfatal[Title/Abstract])

**Table 2:** List of inclusion and exclusion criteria applied to identified papers.

Inclusion Criteria
Cohort studies and cross-sectional design studies; Studies that included custody officers, correctional officers, prison officers, or supervisors/guards of correctional facilities; Studies that provided data on fatal or non-fatal injuries that occurred at work or work-related, including injury rates, number of injuries, prevalence, types of injury, body parts injured, or causes of injury; and Adults of any age, sex, geographical location, and ethnicity that fit the occupation description.
**Exclusion Criteria**
Studies focused on law enforcement officers (including law enforcement officers who underwent custodial duties as part of training); Studies focused on mental health injuries; Full text was not available; Studies that did not include data informing an overall injury profile (e.g., fatal injury focus or specific injury type only); or Studies not in, or translatable to, English.

**Table 3:** Main findings from each included study.

Author (Year)	Demographics	Injury Data Source	Prevalence/Incidence of Non-Fatal Injury/Fatal Injury	Cause of Injury/Fatality	Body Part Injured	Nature of Injury	Critical Appraisal Score & Rating
Konda et al. (2016) [12]	U.S. custody officers between 1998 and 2008. 89% males and 11% females accounted for fatal injuries. 73% males and 27% females accounted for fatal injuries.	Data gathered from Bureau of Labor Statistics Census of Fatal Occupational Injuries.	44 per 1000 FTE for non-fatal. 0.027 per 1000 FTE for fatal. Most common age category for fatal injuries: 45 + years old = 44%. Most common age category for non-fatal injuries: 45+ years old = 22%.	Assaults and violent acts = 38%. Overexertion = 20%. Contact with objects or equipment = 18%. Fatalities: Transportation incidents = 40%. Assaults and violent acts = 40%. (Homicide = 25% and suicide = 15%).	Upper extremity = 30%. Lower extremity = 21%.	Sprains and strains = 30%. Contusions and abrasions = 28%.	7/8 87.5% Good
Holloway-Beth et al. (2016) [15]	LEO (including CO) in Illinois between 1980 and 2008. 74% male and 26% female.	Data gathered through Workers’ Compensation Commission.	15.9 per 1000 FTE for non-fatal. Most common age category: 31–40 years old = 36%.	Falls = 25%. Assaults = 18%. Unspecified = 36%.	Upper extremity = 26%. Lower extremity = 24%.	-	7/8 87.5% Good
Lincoln et al. (2006) [10]	Injuries to correctional staff from weapon attacks in U.S. prisons.	Survey	Weapon attacks 1.0 (0.66–1.28, 95% CI) per 1000 FTE. Mixed-security prisons had the highest rate of staff injuries, 2.0 (1.18–2.81, 95% CI) per 1000 FTE.	-	-	-	3/8 37.5% Poor
Tiesman et al. (2010) [30]	Fatality rates of differently categorised LEO (including CO) in the U.S. between 1992 and 2013.	Data gathered from Bureau of Labor Statistics Census of Fatal Occupational Injuries.	0.030 per 1000 FTE for fatal.	Fatalities: Transportation-related = 40%, followed by homicides = 29% and suicide = 12%.	-	-	8/8 100% Good
Chenpanas and Bir (2017) [32]	Knife fatalities to LEO and CO in the U.S. between 1995 and 2013. Male to female ratio 11:1	Data gathered from autopsy reports.	Age 45–49 years = 33.3%. Age 35–39 years = 25%. Age 40–44 years = 16.7%.	Stab wounds = 70.2%. Slash wounds = 29.8%.	Neck, chest, and shoulders were the most targeted areas.	-	4/8 50% Moderate
Carleton et al. (2017) [31]	Chronic pain among several categories of Canadian public safety personnel (including CO).	Survey	Chronic pain = 45.4%.	-	Lower back pain the most common site of chronic pain = 26.1%.	-	5/8 62.5% Good
Ngwenya (2012) [16]	Prison officers in Australia.	Survey	-	Slips, trips, and falls = 23.2%, followed by contact with objects or equipment = 21.8%, restraints = 11.7%, and assaults = 11.5%.	-	Sprains and strains = 60.2%. Bruising/contusion = 14.8%.	7/8 87.5% Good
Larney and Dolan (2008) [33]	Prison officers in Australia suffering a needlestick injury.	Survey	-	Needlestick injuries = 10%.	-	-	3/8 37.5% Poor

## Data Availability

Data are available from the corresponding author on request.

## Supplementary Materials

The following supporting information can be downloaded at: https://www.mdpi.com/article/10.3390/healthcare12232334/s1, Supplementary File S1: Translations of PubMed Search Strategy for Each Database. Supplementary File S2: Critical Appraisal after Consensus.
