# Supplementary material for: Profiling the Occupational Injuries Sustained by Custody Officers: A Systematic Review
Source: Healthcare (Basel). 2024 Nov 22;12(23):2334. doi: 10.3390/healthcare12232334 (PMC11641701; doi:10.3390/healthcare12232334)
Supplement: Supplementary file 1 [file healthcare-12-02334-s001.zip › Supplementary File S2.pdf]

Supplementary File S2: Critical Appraisal after Consensus.

**Author**	**Item 1**	**Item 2**	**Item 3**	**Item 4**	**Item 5**	**Item 6**	**Item 7**	**Item 8**
**Konda et al. (2016)**	Yes	Yes	Yes	Yes	Yes	No	Yes	Yes
**Holloway-Beth et al. (2016)**	Yes	Yes	Yes	Yes	Yes	No	Yes	Yes
**Lincoln et al. (2006)**	Unclear / No	Yes	Unclear / No	Yes	Unclear / No	No	Yes	No
**Tiesman et al. 2010**	Yes	Yes	Yes	Yes	Yes	Yes	Yes	Yes
**Chenpanas & Bir (2017)**	Yes	Yes	Unclear / No	Yes	Unclear / No	Unclear / No	Yes	No
**Carleton et al. 2017**	No	Yes	Yes	Yes	Yes	Unclear / No	No	Yes
**Ngwenya (2012)**	Yes	Yes	Yes	Yes	Yes	No	Yes	Yes
**Larney & Dolan (2008)**	No	Yes	No	Yes	Unclear /No	No	No	Yes
