# Supplementary material for: Profiling the Occupational Injuries Sustained by Custody Officers: A Systematic Review
Source: Healthcare (Basel). 2024 Nov 22;12(23):2334. doi: 10.3390/healthcare12232334 (PMC11641701; doi:10.3390/healthcare12232334)
Supplement: Supplementary file 1 [file healthcare-12-02334-s001.zip › Supplementary File S1.pdf]

Supplementary File S1: Translations of PubMed Search Strategy for Each Database

**ProQuest**	7/09/23	(MESH.EXACT("Correctional Personnel") OR TI,AB("correctional officer*") OR TI,AB("Custody Officer*") OR TI,AB("Prison Officer*") OR TI,AB("correctional staff") OR TI,AB("Law Enforcement Officer*") OR TI,AB("Correctional Worker*")) AND (MESH.EXACT("Injuries") OR TI,AB(Injury) OR TI,AB(Injured) OR TI,AB(injuries) OR TI,AB(Trauma) OR TI,AB(Wound) OR TI,AB(fatal) OR TI,AB(events) OR TI,AB(nonfatal))
**Embase**	7/09/23	('Correctional Staff'/exp OR 'correctional officer*':ti,ab OR 'Custody Officer*':ti,ab OR 'Prison Officer*':ti,ab OR 'correctional staff':ti,ab OR 'Law Enforcement Officer*':ti,ab OR 'Correctional Worker*':ti,ab) AND ('Injury'/exp OR Injury:ti,ab OR Injured:ti,ab OR injuries:ti,ab OR Trauma:ti,ab OR Wound:ti,ab OR fatal:ti,ab OR events:ti,ab OR nonfatal:ti,ab)
**CINAHL**	7/09/23	((MH "Correctional Facilities Personnel+") OR (TI "correctional officer*" OR AB "correctional officer*") OR (TI "Custody Officer*" OR AB "Custody Officer*") OR (TI "Prison Officer*" OR AB "Prison Officer*") OR (TI "correctional staff" OR AB "correctional staff") OR (TI "Law Enforcement Officer*" OR AB "Law Enforcement Officer*") OR (TI "Correctional Worker*" OR AB "Correctional Worker*")) AND ((MH "Wounds and Injuries+") OR (TI Injury OR AB Injury) OR (TI Injured OR AB Injured) OR (TI injuries OR AB injuries) OR (TI Trauma OR AB Trauma) OR (TI Wound OR AB Wound) OR (TI fatal OR AB fatal) OR (TI events OR AB events) OR (TI nonfatal OR AB nonfatal))
**Sport discus**	7/09/23	((TI "correctional officer*" OR AB "correctional officer*") OR (TI "Custody Officer*" OR AB "Custody Officer*") OR (TI "Prison Officer*" OR AB "Prison Officer*") OR (TI "correctional staff" OR AB "correctional staff") OR (TI "Law Enforcement Officer*" OR AB "Law Enforcement Officer*") OR (TI "Correctional Worker*" OR AB "Correctional Worker*")) AND (DE "Wounds & Injuries" OR (TI "Injury" OR AB "Injury") OR (TI "Injured" OR AB "Injured") OR (TI "injuries" OR AB "injuries") OR (TI "Trauma" OR AB "Trauma") OR (TI "Wound" OR AB "Wound") OR (TI "fatal" OR AB "fatal") OR (TI "events" OR AB "events") OR (TI "nonfatal" OR AB "nonfatal"))
